# Speed and energy optimized quasi-delay-insensitive block carry lookahead adder

**DOI:** 10.1371/journal.pone.0218347

**Published:** 2019-06-21

**Authors:** P. Balasubramanian, D. L. Maskell, N. E. Mastorakis

**Affiliations:** 1 School of Computer Science and Engineering, Nanyang Technological University, Singapore; 2 Department of Industrial Engineering, Technical University of Sofia, Sofia, Bulgaria; Lanzhou University of Technology, CHINA

## Abstract

We present a new asynchronous quasi-delay-insensitive (QDI) block carry lookahead adder with redundant carry (BCLARC) realized using delay-insensitive dual-rail data encoding and 4-phase return-to-zero (RTZ) and 4-phase return-to-one (RTO) handshaking. The proposed QDI BCLARC is found to be faster and energy-efficient than the existing asynchronous adders which are QDI and non-QDI (i.e., relative-timed). Compared to existing asynchronous adders corresponding to various architectures such as the ripple carry adder (RCA), the conventional carry lookahead adder (CCLA), the carry select adder (CSLA), the BCLARC, and the hybrid BCLARC-RCA, the proposed BCLARC is found to be faster and more energy-optimized. The cycle time (CT), which is expressed as the sum of the worst-case times taken for processing the data and the spacer, governs the speed. The product of average power dissipation and CT viz. the power-cycle time product (PCTP) defines the low power/energy efficiency. For a 32-bit addition, the proposed QDI BCLARC achieves the following reductions in design metrics on average over its counterparts when considering RTZ and RTO handshaking: i) 20.5% and 19.6% reductions in CT and PCTP respectively compared to an optimum QDI early output RCA, ii) 16.5% and 15.8% reductions in CT and PCTP respectively compared to an optimum relative-timed RCA, iii) 32.9% and 35.9% reductions in CT and PCTP respectively compared to an optimum uniform input-partitioned QDI early output CSLA, iv) 47.5% and 47.2% reductions in CT and PCTP respectively compared to an optimum QDI early output CCLA, v) 14.2% and 27.3% reductions in CT and PCTP respectively compared to an optimum QDI early output BCLARC, and vi) 12.2% and 11.6% reductions in CT and PCTP respectively compared to an optimum QDI early output hybrid BCLARC-RCA. The adders were implemented using a 32/28nm CMOS technology.

## 1. Introduction

The 2017 edition of the International Roadmap for Devices and Systems [1] suggests that asynchronous design could be a potential solution to address the increasing power/energy consumption of a digital circuit/system. Substantiating this, in [2], a 128-point, 16-bit, radix-8 fast Fourier transform (FFT) processor was implemented in the robust QDI asynchronous design style and it was compared with a conventional synchronous FFT processor implementation, and both these were realized using a 65nm CMOS technology. It was noted that, the QDI FFT processor is 34× more energy-efficient than its synchronous equivalent. The QDI design style is a promising alternative to the synchronous design style, and different types of QDI implementations exist.

QDI circuits are known to be robust to process, voltage, timing and temperature variations [3, 4], which is important to note since the issue of variability [5] is quite common in the nanoelectronics era. Moreover, QDI circuits are less affected by electromagnetic interference compared to synchronous circuits [6]. These properties make QDI circuits preferable for secure applications [7, 8]. Further, QDI circuits and systems are modular [9], and hence they are convenient to reuse or replace thus obviating the need for extensive timing re-runs and analysis. Furthermore, QDI circuits are naturally elastic [10] unlike synchronous circuits, and they are suitable for subthreshold operation [11].

A QDI circuit is the practically realizable delay-insensitive circuit which includes the weakest compromise of the isochronic fork [12]. The isochronic fork assumption implies that all the wires branching out from a node/junction would experience concurrent rising and falling signal transitions. Usually, the isochronic fork assumption is confined to a small circuit area and hence their realization would not be difficult. It has been shown in [13] that QDI circuits are realizable in the nano-electronics regime.

Addition is a fundamental operation in computer arithmetic, which is realized using the adder, and an effective adder design is of interest and importance. This article deals with the high-speed and energy-efficient QDI realization of the adder.

In a latest work [14], several asynchronous implementations of a 32-bit adder were considered and analyzed. QDI full adders based on [15, 16, 17] are strongly indicating (acknowledging) implying that these full adders would wait for the arrival of all the primary inputs and then process them to produce the required primary outputs. When such strong-indication full adders are cascaded to form an N-bit RCA, the RCA would be weakly indicating [14]. The main drawback with this weakly indicating RCA is that a worst-case critical path delay involving N full adders would be encountered for processing the ‘data’ (called ‘forward latency’) and a similar critical path delay would be encountered for processing the ‘spacer’ (called ‘reverse latency’) which affects their speed (CT) and increases their energy (PCTP). The terminologies ‘data’ and ‘spacer’ in the context of RTZ and RTO handshake protocols are explained in Section 3.

Reference [18] yields a weak-indication QDI full adder based on the concept of binary decision diagram, whose sum output would wait for the arrival of all the primary inputs while its carry output need not thus potentially speeding-up the carry propagation. When N instances of the weak-indication full adder of [18] are cascaded to form a QDI RCA, the RCA would be weakly indicating. Although the forward and reverse latencies of a N-bit QDI RCA based on [18] are data-dependent, they would still involve N full adders in the worst-case, which is not optimum from the speed and energy perspectives.

In [19], a biased weak-indication QDI full adder was proposed where the sum output of the full adder is responsible for indicating the arrival of all the primary inputs while the carry output is not. When N weak-indication full adders corresponding to [19] are cascaded, the resulting QDI RCA would encounter a data-dependent forward latency, and a constant reverse latency governed by the sum of the propagation delays of just two full adders. Although the forward latency may be dictated by the sum of the delays of N full adders, the reverse latency would be dictated by the sum of the delays of only two full adders, which is useful for optimizing the speed and energy parameters. It is to be noted that the forward and reverse latencies of N-bit QDI RCAs constructed using the full adders of [20] and [21] are theoretically the same as discussed for [19]. However, [20] presents an improved weak-indication full adder compared to [19], with the carry output logic of the former being better optimized than the latter.

Reference [21] presents an early output QDI full adder whose sum output is responsible for indicating the arrival of all the primary inputs while the carry output is freed from the indication constraint. In general, an early output circuit is able to produce all the primary outputs after receiving a subset of the primary inputs, which may correspond to either data or spacer but not both. An N-bit weak-indication QDI RCA incorporating the early output full adder of [21] would have a forward latency equal to the sum of the delays of N full adders, and a reverse latency equal to the sum of the delays of just two full adders. However, the forward latency of the RCA based on [21] is less compared to the forward latencies of [19] and [20] since the carry output logic of the full adder of [21] is better optimized compared to the carry output logic of the full adders of [19] and [20].

Reference [22] presented early output full adders which when cascaded lead to relative-timed RCAs. Relative-timed RCAs [22] experience a forward latency equivalent to the sum of the delays of N full adders and the optimal constant reverse latency equivalent to the delay of just one full adder. Relative-timed circuits [23] are like early output circuits in that after receiving a subset of the primary inputs (data or spacer), they are able to produce all the primary outputs (data or spacer respectively). However, relative-timed circuits usually incorporate additional timing assumptions with respect to sequencing the arrival of internal signals within the circuit besides the assumption of isochronic forks, which may be rather sophisticated to realize. Relative-timed circuits are not QDI circuits, however they are able to facilitate improvements in the design parameters such as less area, higher speed, and less energy but at the expense of a compromise in the robustness. In contrast, strong-indication, weak-indication and early output QDI circuits are robust.

QDI CLAs have also been discussed in the literature [24, 25, 26, 27] and these correspond to weak-indication or early output type. Among these, [24] presents a full-custom design at the transistor level while [25, 26, 27] present semi-custom designs which correspond to a gate-level synthesis. In general, QDI CLAs are classified into QDI CCLA [25] and QDI BCLAs and BCLARCs [14, 26, 27]. QDI CCLA, BCLAs and BCLARCs tend to have lesser forward latencies compared to the forward latencies of some QDI and relative-timed (non-QDI) RCAs [14]. However, this advantage may be offset by their greater reverse latencies compared to the reverse latencies of QDI and relative-timed RCAs [14]. These observations are also applicable for a comparison made between QDI CSLAs [28] and QDI and relative-timed RCAs [15, 16, 17, 18, 19, 20, 21, 22]. QDI CLAs and CSLAs consume more area compared to the area occupancies of QDI and relative-timed RCAs, as observed in [14].

A QDI BCLA does not incorporate redundant carry output logic [29] while a QDI BCLARC does, and the latter is able to facilitate considerable reductions in forward and reverse latencies and cycle time compared to the former. Hence, QDI BCLARCs are preferable among the category of QDI CLAs. A hybrid QDI BCLARC-RCA architecture, which incorporates an appropriate size RCA in the least significant adder bit positions as a replacement for one or more instances of a sub-BCLARC may enable a further optimization of the design metrics compared to the basic QDI BCLARC architecture. However, this is not guaranteed and should be ascertained case-by-case based on timing analysis. In [14], a hybrid QDI BCLARC-RCA outperformed the QDI RCAs, CSLAs, and other BCLAs and BCLARCs mentioned above in terms of speed and energy.

This article presents a new QDI BCLARC that outperforms all the QDI and non-QDI RCAs, CSLAs, CCLA, BCLAs, BCLARCs and hybrid BCLARC-RCAs described in [14] and [22] in terms of speed (CT) and energy (PCTP). The rest of the article is organized as follows. Section 2 mentions the frequently used acronyms and their expansions for a quick reference. Section 3 discusses the design preliminaries of QDI and non-QDI (relative-timed) asynchronous circuits. Section 4 describes the proposed QDI sub-BCLA block without and with the redundant carry output and the resulting QDI BCLAs, BCLARCs and BCLARC-RCAs by considering an example 32-bit addition. Section 5 presents the design metrics for several 32-bit QDI and non-QDI asynchronous adders corresponding to 4-phase RTZ and 4-phase RTO handshaking, and they are compared. Finally, Section 6 draws the conclusions.

## 2. Acronyms and Expansions

Widely used acronyms and their expansions are given below for a ready reference.

- CLA–Carry Lookahead Adder
- BCLA–Block CLA
- BCLARC–BCLA with Redundant Carry
- BCLG–Block Carry Lookahead Generator
- BCLGRC–BCLG with Redundant Carry
- CCLA–Conventional CLA
- CSLA–Carry Select Adder
- CT–Cycle Time
- PCTP–Power-Cycle Time Product
- RCA–Ripple Carry Adder
- QDI–Quasi-delay-insensitive
- RTO–Return-To-One
- RTZ–Return-To-Zero

## 3. QDI and Non-QDI Circuits–A Background

The design fundamentals of QDI and non-QDI (i.e., relative-timed) asynchronous circuits are discussed in this section to provide a background.

### 3.1. Data encoding, handshaking, and timing parameters

**Fig 1:**
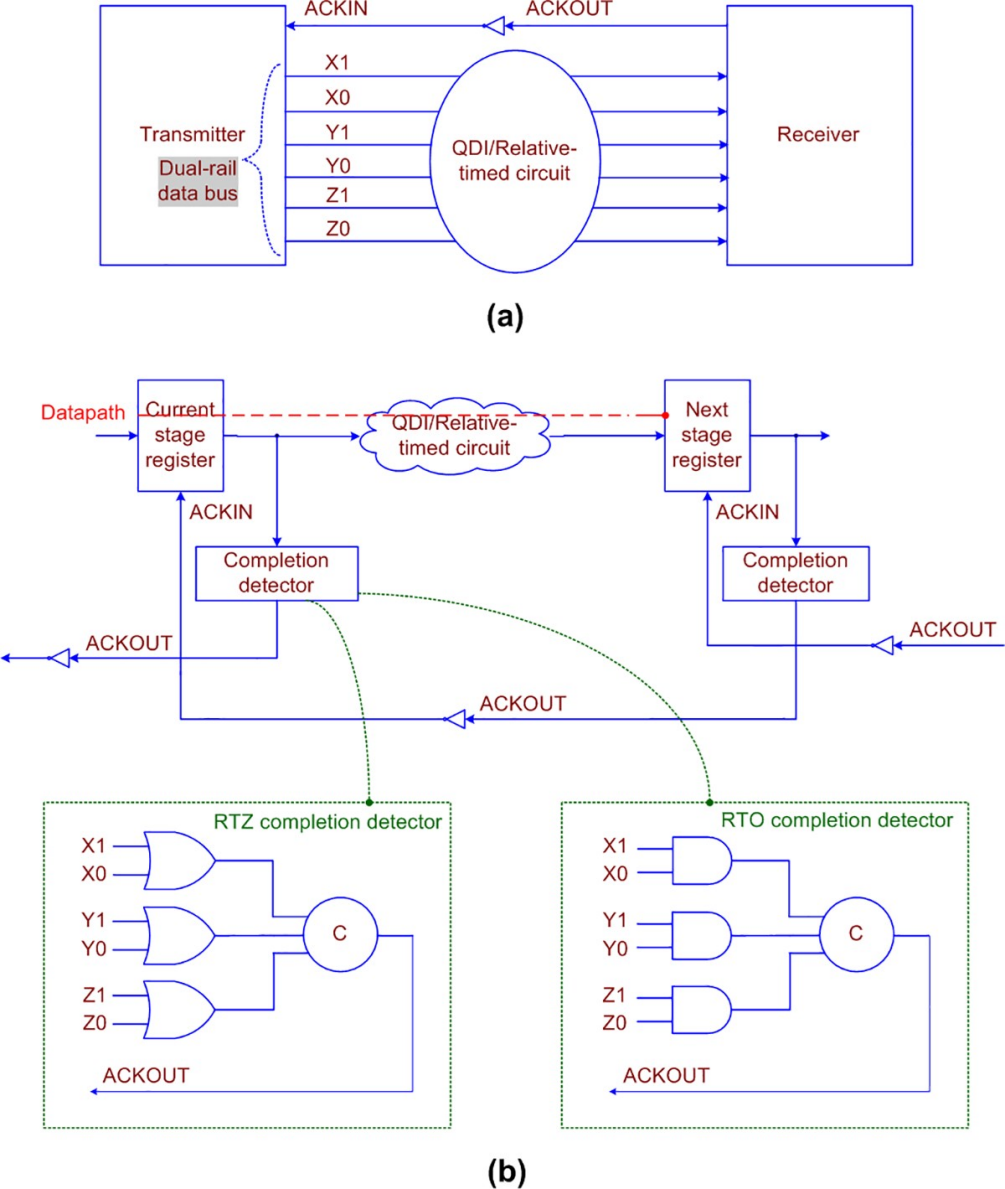
(a) Transmitter-Receiver analogy of a QDI/non-QDI (relative-timed) asynchronous circuit stage, and (b) technical schematic portraying the example RTZ and RTO completion detectors for the presumed dual-rail data bus comprising inputs (X1, X0), (Y1, Y0) and (Z1, Z0). The OR and AND gates used in the RTZ and RTO completion detectors are duals of each other. The datapath is highlighted by the red dashed line in (b).

The general schematic of a QDI or a relative-timed circuit stage employing delay-insensitive data encoding and a 4-phase handshaking is shown in Fig 1A, which corresponds to the transmitter-receiver analogy. The technical schematic is shown in Fig 1B.

In Fig 1B, the current stage and next stage registers are analogous to the transmitter and the receiver, shown in Fig 1A, and a QDI or a relative-timed circuit is sandwiched between the current stage and the next stage register banks. The register bank comprises a series of registers, with one register allotted for each of the rails of a dual-rail encoded data input. A register implies a 2-input Muller C-element [30]. The C-element will output 1 or 0 if all its inputs are 1 or 0 respectively. If the inputs to a C-element are not identical then the C-element would retain its existing steady-state. The circles with the marking ‘C’ represent the C-elements in the figures.

In Fig 1, (X1, X0), (Y1, Y0) and (Z1, Z0) represent the dual-rail encoded primary inputs of the corresponding single-rail inputs X, Y and Z. According to delay-insensitive dual-rail data encoding and the 4-phase RTZ handshaking [9], an input W is encoded as (W1, W0) where W = 1 is represented by W1 = 1 and W0 = 0, and W = 0 is represented by W0 = 1 and W1 = 0. Both these assignments are called *data*. The assignment W1 = W0 = 0 is called the *spacer*, and the assignment W1 = W0 = 1 is deemed illegal since the coding scheme should be complete [31] and unordered [32] to maintain the delay-insensitivity.

The application of input data to a QDI or relative-timed circuit which adheres to the 4-phase RTZ handshaking follows the sequence: *data-spacer-data-spacer*, and so forth. It may be noted that the application of data is followed by the application of the spacer, which implies that there is an interim RTZ phase between the successive applications of input data. The interim RTZ phase ensures a proper and robust data communication i.e., handshaking between the transmitter and the receiver. The RTZ handshake protocol is specified by the following four steps:

- First, the dual-rail data bus specified by (X1, X0), (Y1, Y0) and (Z1, Z0) assumes the spacer, and therefore the acknowledgment input (ACKIN) is equal to binary 1. After the transmitter transmits a data, this would cause rising signal transitions i.e., binary 0 to 1 to occur on one of the dual rails of the entire dual-rail data bus
- Second, the receiver would receive the data sent and drive the acknowledgment output (ACKOUT) to 1. ACKIN is the Boolean complement of ACKOUT and vice-versa
- Third, the transmitter waits for ACKIN to become 0 and would subsequently reset the entire dual-rail data bus, i.e., the dual-rail data bus assumes the spacer again
- Fourth, after an unbounded (but a finite and positive) time duration, the receiver would drive ACKOUT to 0 and then ACKIN would assume 1. With this, a single data transaction is said to be completed and the asynchronous circuit is permitted to start the next data transaction

According to dual-rail data encoding and the 4-phase RTO handshaking [33], an input V is encoded as (V1, V0) and V = 1 is represented by V1 = 0 and V0 = 1, and V = 0 is represented by V0 = 0 and V1 = 1. Both these assignments are called *data*. The assignment V1 = V0 = 1 is called the *spacer*, and the assignment V1 = V0 = 0 is deemed illegal to maintain the delay-insensitivity.

The application of input data to a QDI or relative-timed circuit conforming to the 4-phase RTO handshaking follows the sequence: *spacer-data-spacer-data*, and so forth. It may be noted that there is an interim RTO phase between the successive applications of input data. The interim RTO phase ensures a proper and robust data communication between the transmitter and the receiver. The RTO handshaking process is specified by the following four steps:

- First, ACKIN is equal to binary 1. After the transmitter transmits the spacer, this would cause rising signal transitions i.e., binary 0 to 1 to occur on all the rails of the dual-rail data bus
- Second, the receiver would receive the spacer sent and drive ACKOUT to 1
- Third, the transmitter waits for ACKIN to become 0 and would then transmit the data through the dual-rail data bus
- Fourth, after an unbounded (but a finite and positive) time duration, the receiver would drive ACKOUT to 0 and subsequently ACKIN would assume 1. With this, a single data transaction is said to be completed and the asynchronous circuit is permitted to start the next data transaction

In a QDI or relative-timed circuit, the time taken to process the data in the datapath, highlighted by the red dashed line in Fig 1B, is called *forward latency*, and the time taken to process the spacer is called *reverse latency*. Since there is an intermediate RTZ or RTO phase between the application of two input data sequences, the *cycle time* is expressed as the sum of forward and reverse latencies. The cycle time of a QDI or a relative-timed asynchronous circuit is the equivalent of the clock period of a synchronous circuit. The cycle time governs the speed at which new data can be input to an asynchronous circuit.

The gate-level details of example completion detectors corresponding to RTZ and RTO handshaking is shown at the bottom of Fig 1B, within the dotted green boxes. The completion detector indicates i.e., acknowledges the receipt of all the primary inputs given to an asynchronous circuit stage. In the case of 4-phase RTZ handshaking, ACKOUT is produced by using a 2-input OR gate to combine the respective dual rails of each encoded primary input and synchronizing the outputs of all the 2-input OR gates using a C-element or a tree of C-elements. In the case of 4-phase RTO handshaking, ACKOUT is produced by using a 2-input AND gate to combine the respective dual rails of each encoded primary input and then synchronizing the outputs of all the 2-input AND gates using a C-element or a tree of C-elements.

### 3.2. QDI circuits

**Fig 2:**
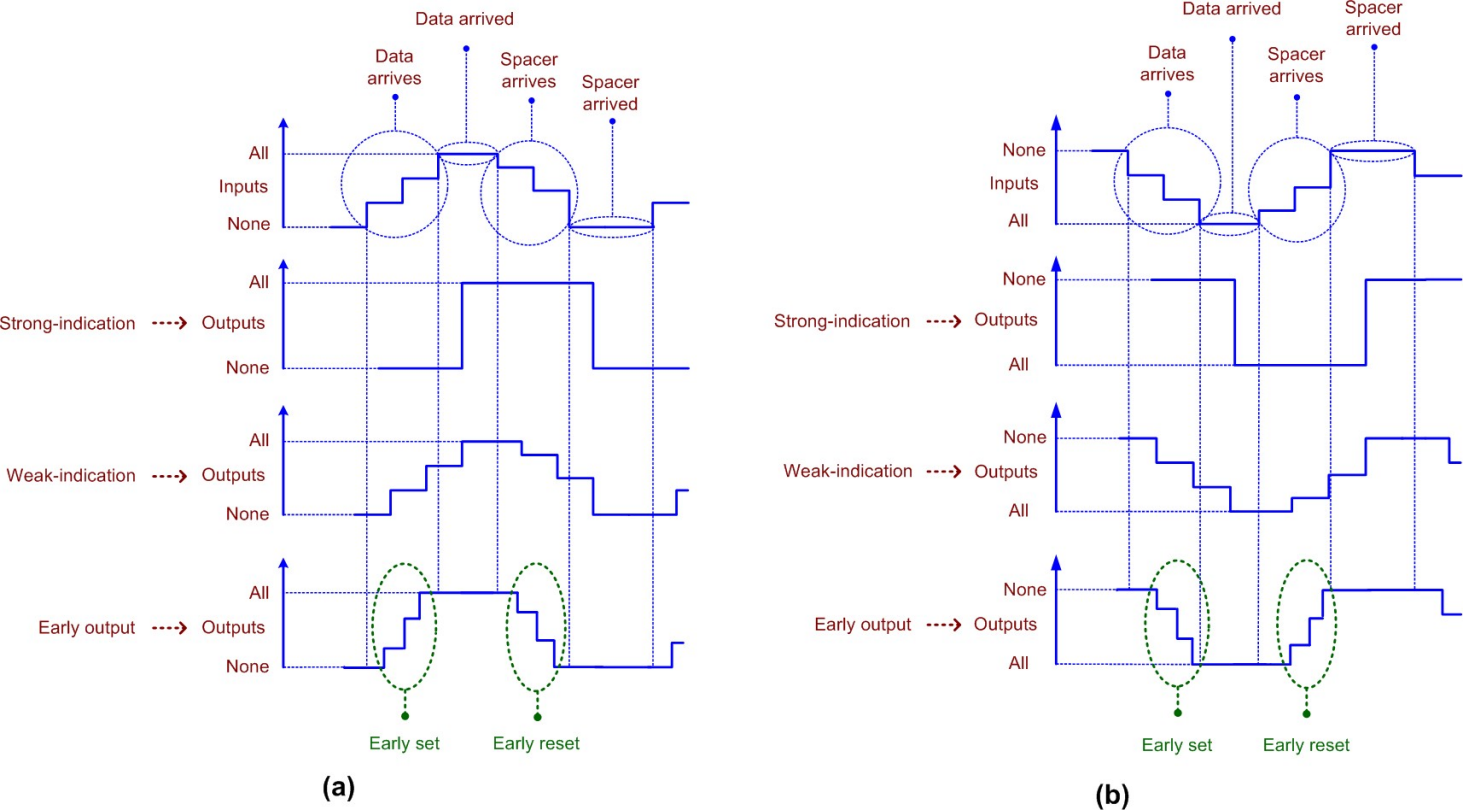
Input-output timing relation of different QDI circuits corresponding to (a) RTZ handshaking, and (b) RTO handshaking. Early set and reset behaviors of the early output circuit type are highlighted by the dotted green ovals in (a) and (b).

QDI circuits are classified into three types as strong-indication [34], weak-indication [34], and early output [35] circuits. The input-output timing relations of QDI circuits are illustrated by the representative timing diagrams shown in Fig 2A and 2B with respect to RTZ and RTO handshaking.

Strong-indication circuits would wait to receive all the primary inputs (data and spacer), and after receiving them would process to produce the required primary outputs (data and spacer respectively). On the other hand, weak-indication circuits can produce all but one of the primary outputs after receiving a subset of the primary inputs. Nevertheless, only after receiving the last primary input, they would produce the last primary output.

A connection of strong-indication sub-circuits may not result in a strong-indication circuit; rather, a weak-indication circuit may result. For example, if two strong-indication full adders are connected, a weak-indication 2-bit RCA would result. This is because if all the inputs to one of the full adders are provided, the corresponding sum and carry output bits of that full adder could be produced regardless of the arrival/non-arrival of the inputs to the other full adder in the RCA. However, only after all the inputs to the other full adder are provided, its corresponding sum and carry output bits would be produced. This scenario is characteristic of weak-indication.

For implementing arithmetic functions, weak-indication is preferable to strong-indication and this is due to the following reasons: i) strong-indication arithmetic circuits tend to encounter worst-case forward and reverse latencies for the application of data and spacer, and therefore the cycle time of strong-indication arithmetic circuits is always the maximum (worst-case timing), ii) weak-indication arithmetic circuits may encounter data-dependent forward and reverse latencies or a data-dependent forward latency and a constant reverse latency, and so the cycle times of weak-indication arithmetic circuits are usually less compared to strong-indication arithmetic circuits.

An early output circuit is however more relaxed compared to strong- and weak-indication circuit counterparts. After receiving a subset of the primary inputs (data or spacer), an early output circuit can produce all the primary outputs (data or spacer respectively). This implies the late arriving primary inputs may not be acknowledged by the circuit. However, this is not a cause for concern because isochronic fork assumptions are imposed on all the primary inputs, and all the primary inputs are given to the completion detector that precedes the early output circuit, as seen in Fig 1B. Hence, the acknowledgment of the late arriving primary inputs by the completion detector also implies the receipt of those primary inputs by the asynchronous circuit. Thus, the problem of wire orphan(s) i.e., unacknowledged signal transitions on the wire(s) due to the late arrival of primary input(s) is overcome by the assumption of isochronic forks, which is imposed on all the primary inputs.

Either the data may be produced early, or the spacer may be produced early in an early output circuit and not both. Accordingly, an early output circuit is categorized as early set or early reset kind. The early set and reset behaviors of early output circuits are highlighted by the dotted green ovals in Fig 2A and 2B. An early output RCA is preferable to a strong-indication and a weak-indication RCA for achieving better optimizations in speed and power/energy [14]. In general, an early output circuit can achieve enhanced optimizations in the design metrics compared to strong- and weak-indication counterparts.

In a QDI circuit, the logic decomposition should be performed safely [36]. Safe QDI logic decomposition [17] is essential to avoid the problem of gate orphans, which are unacknowledged signal transitions occurring on the intermediate gate output(s). For an illustration of gate and wire orphans, the interested reader is referred to [37]. However, we discuss about orphans in the following section.

The signal transitions will have to occur monotonically in a QDI circuit from the first logic level, which receives the primary inputs, up to the last logic level, which produces the primary outputs [38]. The signal transitions should either be seen rising or falling throughout an entire QDI circuit. In general, the signal transitions will be rising (i.e., binary 0 to 1) for the application of data, and falling (i.e., binary 1 to 0) for the application of spacer in a QDI circuit that corresponds to RTZ handshaking. On the other hand, the signal transitions will be rising for the application of spacer and falling for the application of data in a QDI circuit that corresponds to RTO handshaking.

For monotonicity of signal transitions, the monotonic cover constraint [9] should be incorporated into a QDI logic description. For example, if a QDI logic function is expressed in the sum-of-products form, only one product term should be activated for the application of input data, i.e., the product terms comprising the sum-of-products expression of a QDI logic function should be mutually orthogonal (also called disjoint), i.e., the logical conjunction of any two product terms in a QDI logic function should yield zero. Thus, a QDI logic function is ideally expressed in the disjoint sum-of-products form [39], which would consist of mutually disjoint products to satisfy the monotonic cover constraint. An example illustration of the monotonic cover constraint is given in Section 2.2 of [14], and an interested reader may refer to the same for details. Embedding the monotonic cover constraint and performing safe QDI logic decomposition are central to the correct implementation of a QDI circuit.

Incorporating the monotonic cover constraint in a QDI logic function would ensure the activation of just one signal path from a primary input to a primary output for the application of an input data. This is useful to facilitate the proper acknowledgment of signal transitions throughout an entire QDI circuit, thus avoiding the likelihood of any gate orphan occurrence(s). Gate orphans are troublesome unlike wire orphans as they may affect the robustness of a QDI circuit and if they are imminent, restricting them from affecting the circuit robustness may require incorporating additional timing assumptions which are likely to be sophisticated, and may be difficult to realize [22].

### 3.3. Relative-timed (Non-QDI) circuits

Relative-timed circuits [23] are not QDI circuits although they may embed the monotonic cover constraint and adopt safe QDI logic decomposition for their physical realization. This is because relative-timed circuits tend to incorporate extra timing assumptions (in addition to the assumption of isochronic forks), to eliminate any potential problem due to gate orphan(s). Usually, the extra timing assumptions are related to the delayed arrival of some internal input signals, which are subject to a specific time bound. If the timing assumptions are upheld in a relative-timed circuit the circuit would appear to be QDI, and supposing they are violated, the circuit would not be QDI. Relative-timed circuits are early output circuits; however, they are non-QDI unlike the latter. A couple of relative-timed RCAs were presented in [22], which were realized using early output full adders. Relative-timed circuits are seen to be competitive to early output QDI circuits as they could pave the way for enhanced optimizations of the design metrics compared to strong-indication, weak-indication and early output QDI circuits but at the expense of a compromise in the robustness. Hence, only strong-indication, weak-indication and early output QDI circuits are robust and are guaranteed to be gate-orphan free.

## 4. Proposed QDI BCLA and QDI BCLARC

### 4.1. Generic CCLA and BCLA architectures–A brief comparison

In general, an N-bit CCLA is constructed by cascading (N/M) M-bit CCLAs where N modulo M equals 0 [40]. The M carry outputs of a M-bit CCLA are produced by lookahead based on the corresponding generate and propagate functions and also the carry input. Of the M carry outputs, excepting the most significant lookahead carry output, the remaining (M–1) carry outputs are XOR-ed with the corresponding propagate functions to produce the respective sum output bits. The most significant lookahead carry output produced by a M-bit CCLA is propagated to the next M-bit CCLA to serve as its carry input, which is utilized to produce its corresponding sum and carry output bits.

An N-bit BCLA [41], also called the section-carry based CLA [25], is also realized using (N/M) M-bit BCLAs where N modulo M equals 0. However, a M-bit BCLA comprises a M-bit BCLG, three full adders, and a final 3-input XOR function. A M-bit BCLG produces just one carry output by lookahead based on the propagate and generate functions and the carry input, which is then propagated to the successive M-bit BCLA to serve as its carry input. The carry input to an M-bit BCLA along with its corresponding augend and addend inputs are processed by a kind of sub-RCA which is also of size M-bits that features a cascade of (M–1) full adders and a final 3-input XOR function to produce the respective sum output bits. Hence, the intermediate carries in a M-bit BCLA are not produced by lookahead; rather they are produced in a ripple-carry fashion.

### 4.2. QDI BCLA and BCLARC architectures

**Fig 3:**
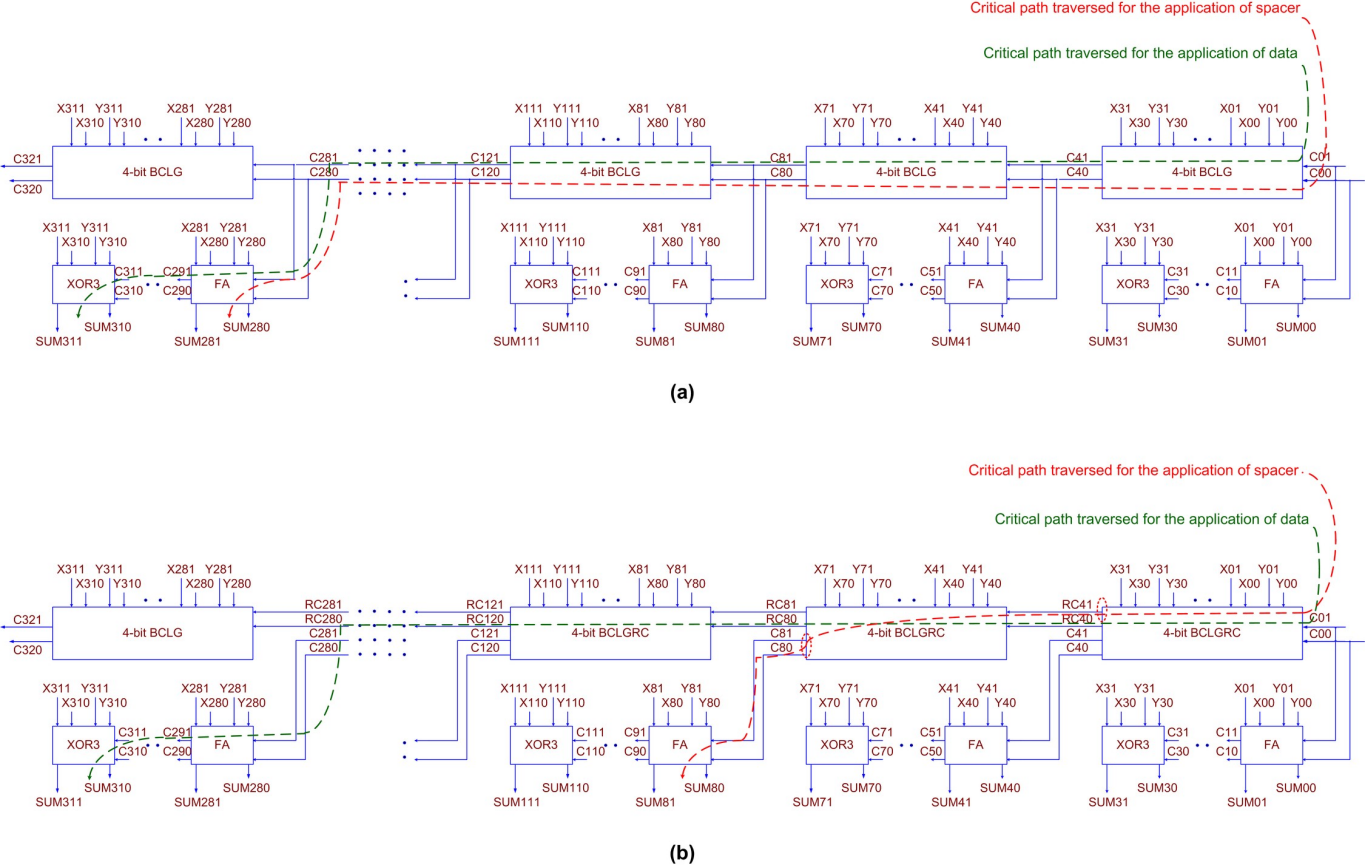
(a) 32-bit QDI BCLA, and (b) 32-bit QDI BCLARC. The architectures remain the same for RTZ and RTO handshaking. The critical paths traversed for the application of data and spacer also remain the same for RTZ and RTO handshaking. One non-redundant lookahead carry output is produced by each 4-bit QDI BCLG in (a), whereas a non-redundant lookahead carry output and a redundant lookahead carry output is produced by each 4-bit QDI BCLGRC in (b). FA refers to the full adder and XOR3 refers to the 3-input XOR function, and both these belong to (QDI) early output type.

The architectures of QDI BCLA and QDI BCLARC for an example 32-bit addition are shown in Fig 3. We consider the 32-bit addition here so as to facilitate a straightforward comparison with the recent published literature [14, 22].

Fig 3A shows a 32-bit BCLA that comprises eight 4-bit BCLGs, 24 full adders, and eight 3-input XOR (XOR3) functions. Fig 3B shows a 32-bit BCLARC that comprises the most significant 4-bit BCLG, seven less significant 4-bit BCLGRCs, 24 full adders and eight XOR3 functions. In Fig 3A and 3B, (X01, X00) and (Y01, Y00) denote the least significant dual-rail encoded augend and addend inputs, and (X311, X310) and (Y311, Y310) represent the most significant dual-rail encoded augend and addend inputs. The dual-rail encoded carry input and output are denoted by (C01, C00) and (C321, C320) respectively, and the carry input can be set to 0 for RTZ handshaking and set to 1 for RTO handshaking. The critical datapaths traversed for the application of data and spacer in the adders are highlighted by the green and red dashed lines in Fig 3A and 3B respectively. It can be noticed in Fig 3 that the 4-bit BCLG, the 4-bit BCLGRC, the full adder, and the XOR3 function form the basic building blocks of the QDI BCLA and the QDI BCLARC.

This work presents the novel and efficient design of a 4-bit BCLG and BCLGRC, which are QDI. The 4-bit BCLG and BCLGRC form the heart of the 4-bit BCLA and the 4-bit BCLARC, which eventually form the building blocks for the QDI BCLA and the QDI BCLARC. QDI realizations of the full adder and the XOR3 function, which were discussed in our previous work [14], have been utilized here to realize the BCLA and the BCLARC. The XOR3 function is referred to as the sum logic in [14].

**Fig 4:**
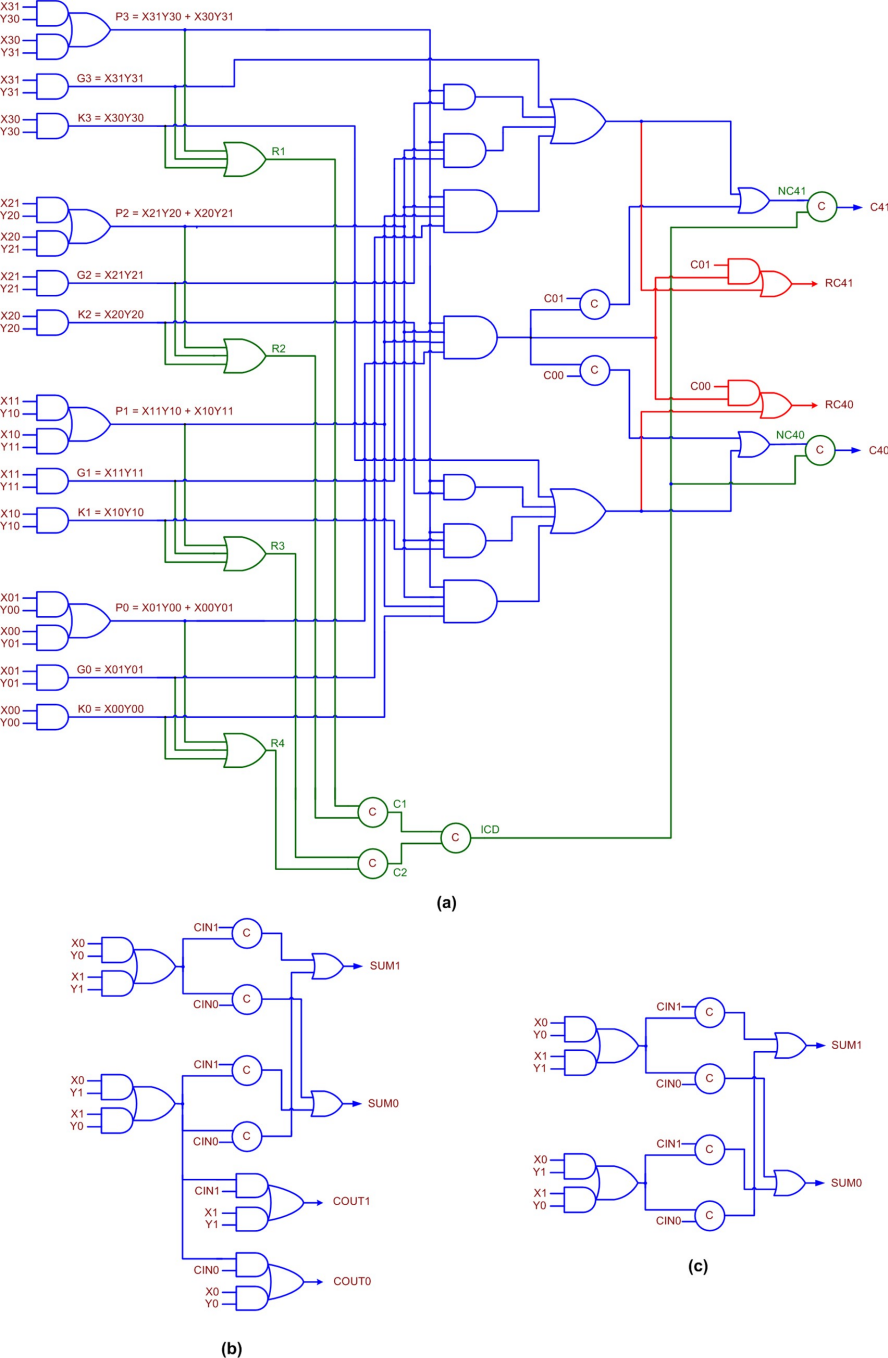
(a) Proposed QDI 4-bit BCLG/BCLGRC, (b) early output QDI full adder, and (c) early output QDI XOR3 function. All the circuits correspond to 4-phase RTZ handshaking. Note that if the circuit portion shown in red is omitted in (a), it is called 4-bit BCLG; if the circuit portion shown in red is included in (a), it is called 4-bit BCLGRC–this interpretation of 4-bit BCLG and 4-bit BCLGRC is also applicable to Fig 5(a). The circuit portion shown in green lines signifies the internal completion detection.

**Fig 5:**
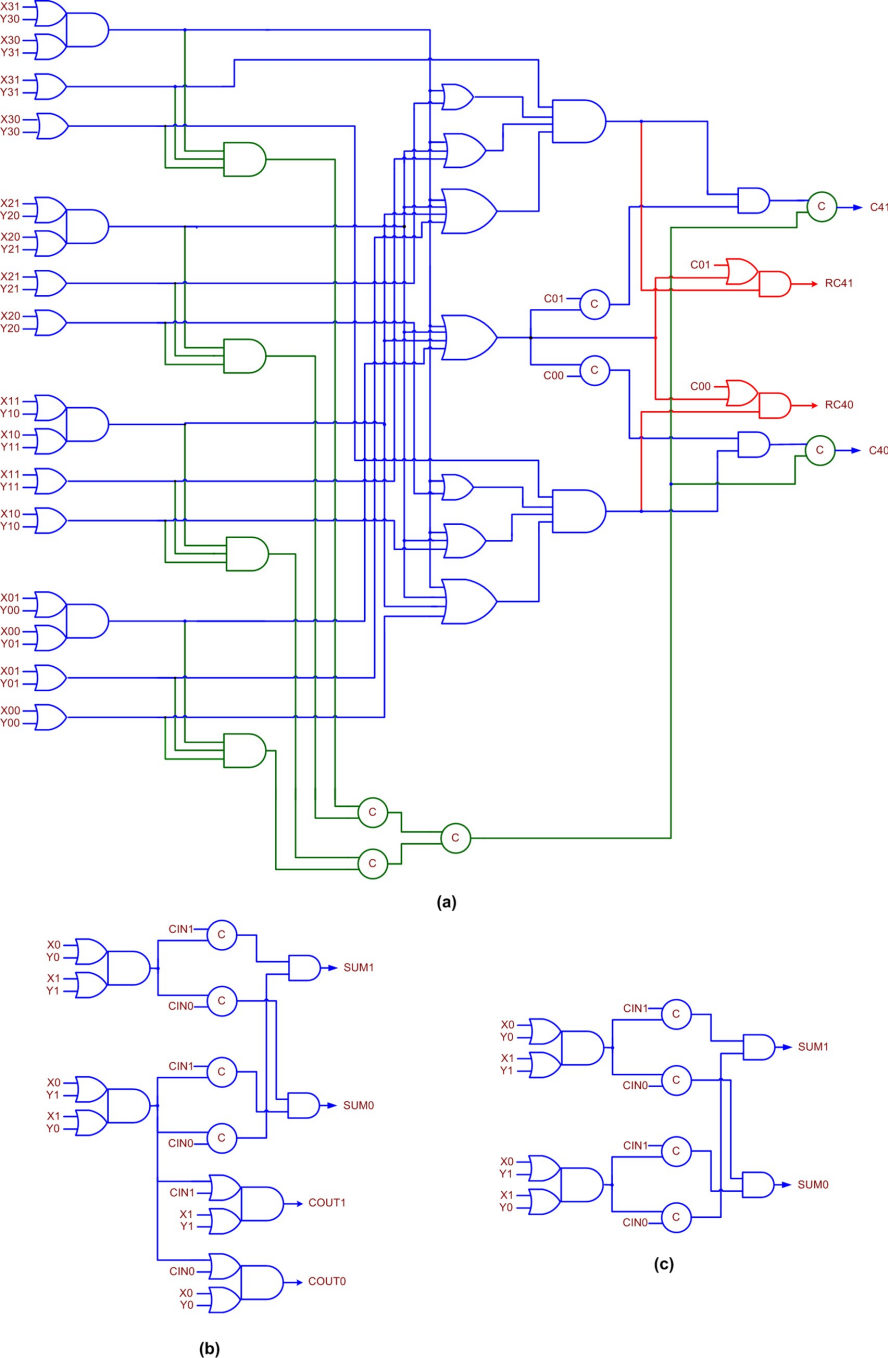
(a) Proposed QDI 4-bit BCLG/BCLGRC, (b) early output QDI full adder, and (c) early output QDI XOR3 function. All the circuits correspond to 4-phase RTO handshaking. The circuit portion shown in green lines signifies the internal completion detection.

Gate-level realizations of the 4-bit QDI BCLG/BCLGRC, the early output QDI full adder, and the early output QDI XOR3 function corresponding to RTZ handshaking are shown in Fig 4A, 4B and 4C respectively. The equivalent gate-level circuits corresponding to RTO handshaking are depicted in Fig 5A, 5B and 5C respectively. It is proved in [42] that any asynchronous circuit corresponding to RTZ handshaking can be transformed into that corresponding to RTO handshaking and vice-versa by replacing the logic gates by their respective duals while retaining the C-elements and their respective inputs as such. We shall describe the basic building blocks shown in Fig 4 which correspond to RTZ handshaking, and the discussion will be applicable to those in Fig 5, which correspond to RTO handshaking.

Fig 4A shows the proposed 4-bit QDI BCLG/BCLGRC. (C01, C00) represents the dual-rail carry input, (C41, C40) represents the dual-rail lookahead carry output, and (RC41, RC40) is the *redundant* dual-rail lookahead carry output, which is logically equivalent to (C41, C40). The equations for (C41, C40) are given in (1) and (2), which are applicable for (RC41, RC40).

C41=G3+P3G2+P3P2G1+P3P2P1G0+P3P2P1P0C01 (1)

C40=K3+P3K2+P3P2K1+P3P2P1K0+P3P2P1P0C00 (2)

In (1) and (2), G3 to G0 represent the carry-generate functions, P3 to P0 represent the carry-propagate functions, and K3 to K0 represent the carry-kill functions. The logic expressions for these functions are given in Fig 4A. The carry-propagate, carry-generate, and carry-kill functions are mutually orthogonal, which implies that only one of these functions corresponding to a set of primary inputs will be activated for the application of an input data. For example, referring to Fig 4A, either G3 or P3 or K3 will alone assume 1 during a data phase and the rest will continue to maintain 0 from the earlier RTZ phase. Eqs (1) and (2) are thus inherently in the disjoint sum-of-products form.

Note that in Figs 4A and 5A, if the circuit portion shown in red is omitted, they represent the ‘4-bit QDI BCLG’, and if the circuit portion shown in red is included, they represent the ‘4-bit QDI BCLGRC’. The circuit portion shown in green lines in Figs 4A and 5A signifies the internal completion detection, which is crucial to ensure freedom from gate orphan(s). The QDI BCLG features only the lookahead carry output (C41, C40), and the QDI BCLGRC features the extra redundant lookahead carry output (RC41, RC40). The proposed 4-bit BCLG and 4-bit BCLGRC belong to the early output type; the BCLG and the BCLGRC will wait for the arrival of required data on the primary inputs to produce the corresponding primary outputs. However, after the assumption of spacer by a subset of the primary inputs, all the primary outputs could assume the spacer.

In Fig 4A, R1, R2, R3, R4, C1, C2, ICD, NC41 and NC40 represent the intermediate outputs. These internal outputs manifest in Fig 5A as well. Each set of the respective carry-generate, carry-propagate and carry-kill functions (for example, G3, P3 and K3) are OR-ed in Fig 4A (AND-ed in Fig 5A) and their outputs viz. R1 to R4 are given to a C-element tree. The output of the C-element tree is denoted as ICD, which is the output of the internal completion detector. NC41 and NC40 are equivalent to C41 and C40. But NC41 and NC40 are synchronized with ICD to produce C41 and C40. This is to ensure that when C41 and C40 are produced all the internal data processing within the 4-bit BCLG/BCLGRC is completed and all the internal outputs have settled to the correct steady-state. Ensuring internal completion detection is necessary for the proposed BCLG/BCLGRC to guarantee that they are QDI.

To illustrate the importance of and the need for internal completion detection in Fig 4A (and Fig 5A), let us assume that P3 = P2 = P1 = G0 = 1 after an RTZ phase. As a result, NC41 would assume 1. Also, R1 = R2 = R3 = R4 = 1. Therefore, C1 = C2 = 1 and ICD = 1. Since NC41 = ICD = 1, C41 = 1 and C40 = 0. Subsequently, in the next RTZ phase, let us assume that only P3, P2 and P1 have become 0 and G0 is still 1. Given this, NC41 will assume 0. Supposing, NC41 was used to represent C41, this will incorrectly convey that the BCLG/BCLGRC has assumed the spacer although the internal data processing has not been completed because G0 has not yet become 0. This violates the QDI principle because in a QDI circuit, the production of primary outputs should unambiguously confirm the receipt of the primary inputs and the completion of internal computation within the circuit for the processing of data and spacer. This will avoid the likelihood of any gate orphan(s), which would occur if the output(s) of intermediate gate(s) remain unacknowledged.

### 4.3. Cycle time calculation of proposed QDI BCLA and BCLARC

It would be useful to analyze the (worst-case) CTs of the proposed QDI BCLA and BCLARC to gain an insight into which of these architectures would be beneficial in terms of the speed prior to physical realization. To estimate the CT, the estimation of forward and reverse latencies is essential since CT is the summation of forward and reverse latencies.

#### 4.3.1. Cycle time of QDI BCLA

To theoretically estimate the (worst-case) CT of the proposed QDI BCLA that corresponds to RTZ handshaking, let us consider Fig 3A and Fig 4. Let T_BCLG_, T_FA_ and T_XOR3_ represent the propagation delays of the QDI early output 4-bit BCLG, the full adder, and the XOR3 function respectively, which are shown in Fig 4. Let the propagation delays of the least significant 4-bit BCLG and the intermediate 4-bit BCLG be denoted as T_BCLG_LS_ and T_BCLG_INT_. Given these, the forward latency of the 32-bit QDI BCLA (FL_BCLA_RTZ_) shown in Fig 3A that corresponds to RTZ handshaking, which incorporates the building blocks of Fig 4, is expressed by (3). In (3), the last term on the right-side represents the propagation delay of the input register (T_Register_), which is the propagation delay of the 2-input C-element since the C-element represents the register.

$${FL}_{BCLA\_RTZ}=T_{XOR3}+(3\times T_{FA})+(6\times T_{BCLG\_INT})+T_{BCLG\_LS}+T_{Register}$$ (3)

Referring to Fig 4A, the longest (critical) datapath is traversed in the least significant BCLG which involves an AO22 complex gate, a 3-input OR gate, and three 2-input C-elements. As in the previous works, the 2-input C-element was custom-realized based on a 32/28nm CMOS technology [43] by modifying the AO222 complex gate realization by introducing feedback which required 12 transistors. Besides the C-element, all the other gates in the cell library [43] were directly utilized. In the subsequent intermediate BCLGs, the datapath traversal would encounter relatively fewer gates which involves a 2-input C-element, a 2-input OR gate, and a final 2-input C-element. The datapath traversal in the full adder would involve an AO22 gate, and the datapath traversal via the XOR3 function would involve a 2-input C-element and a 2-input OR gate.

With T_AO22_, T_OR3_, T_CE2_ and T_OR2_ representing the propagation delays of an AO22 complex gate, a 3-input OR gate, a 2-input C-element, and a 2-input OR gate respectively, (3) is expanded and given by (4). Note that there is a one-to-one correspondence between the terms present on the right-side of (3) and (4).

$${FL}_{BCLA\_RTZ}=(T_{CE2}+T_{OR2})+(3\times T_{AO22})+6\times (2\times T_{CE2}+T_{OR2})+(T_{AO22}+T_{OR3}+3\times T_{CE2})+T_{CE2}$$ (4)

Let the reverse latency of the QDI BCLA shown in Fig 3A that corresponds to RTZ handshaking be denoted as RL_BCLA_RTZ_ which is expressed by (5).

$${RL}_{BCLA\_RTZ}=T_{FA}+(6\times T_{BCLG\_INT})+T_{BCLG\_LS}+T_{Register}$$ (5)

Compared to (3), the processing of the spacer in the QDI BCLA involves fewer gates, i.e., two full adders and one XOR3 less. This is because the least significant full adder present in the most significant 4-bit BCLA of Fig 3A would wait for the arrival of the carry input (C281, C280) to process it to produce the sum output bit (SUM281, SUM280). Referring to Fig 4B, the carry outputs of all the full adders can be produced early and when they are given as the carry inputs for the successive full adders in the cascade, the sum outputs of those full adders could be produced simultaneously. This time delay is less compared to the reverse latency of the QDI BCLA shown in Fig 3A. Thus, (5) is expanded and given as (6), and there is a one-to-one correspondence between the terms present on the right-side of (5) and (6).

$${RL}_{BCLA\_RTZ}=(T_{CE2}+T_{OR2})+6\times (2\times T_{CE2}+T_{OR2})+(T_{AO22}+T_{OR3}+{3\times T}_{CE2})+T_{CE2}$$ (6)

The CT of the QDI BCLA (Fig 3A) can be calculated by substituting the propagation delays of minimum-size gates present in the cell library in (4) and (6), and then adding up the forward and reverse latencies. Based on the theoretical calculations, the forward and reverse latencies of the 32-bit QDI BCLA are found to be 2.583ns and 2.367ns, resulting in a CT of 4.95ns for RTZ handshaking.

The detailed expressions for forward and reverse latencies corresponding to RTO handshaking are given by (7) and (8) with reference to Fig 3A and Fig 5. Eqs (7) and (8) are deduced by replacing the propagation delays of the gates mentioned in (4) and (6) with the propagation delays of their dual gates, however, with the exception of T_CE2_, which is retained as such. This is because the 2-input C-elements and their respective inputs are retained as such while transforming a circuit corresponding to RTZ handshaking into that that corresponds to RTO handshaking [42].

$${FL}_{BCLA\_RTO}=(T_{CE2}+T_{AND2})+(3\times T_{OA22})+6\times (2\times T_{CE2}+T_{AND2})+(T_{OA22}+T_{AND3}+3\times T_{CE2})+T_{CE2}$$ (7)

$${RL}_{BCLA\_RTO}=(T_{CE2}+T_{AND2})+6\times (2\times T_{CE2}+T_{AND2})+(T_{OA22}+T_{AND3}+{3\times T}_{CE2})+T_{CE2}$$ (8)

Based on (7) and (8), the forward and reverse latencies of the 32-bit QDI BCLA, shown in Fig 3A, are calculated to be 2.842ns and 2.632ns, resulting in a CT of 5.474ns for RTO handshaking.

#### 4.3.2. Cycle time of QDI BCLARC

To theoretically estimate the (worst-case) CT of the proposed QDI BCLARC that corresponds to RTZ handshaking, let us consider Fig 3B and Fig 4. In Fig 3B, one most significant 4-bit BCLA and seven less significant 4-bit BCLARCs are used. The use of the 4-bit BCLA for the most significant adder nibble position is because only one (non-redundant) lookahead carry output has to be produced which represents the carry overflow.

Starting from the least significant 4-bit BCLARC, each 4-bit BCLARC produces a non-redundant lookahead carry output and a redundant lookahead carry output. The redundant lookahead carry output of a 4-bit BCLGRC is propagated to the successive 4-bit BCLGRC (or 4-bit BCLG) as its carry input, whereas the non-redundant lookahead carry output is propagated to a cascade of three full adders and an XOR3 present in the successive 4-bit BCLARC (or 4-bit BCLA).

Referring to Fig 4A, the critical datapath would be traversed in the least significant 4-bit BCLGRC involving an AO22 complex gate, a 4-input AND gate, a 4-input OR gate, and an AO21 complex gate. In the subsequent intermediate 4-bit BCLGRCs, the datapath traversal would involve just one AO21 complex gate.

The forward latency of the BCLARC corresponding to RTZ handshaking (FL_BCLARC_RTZ_), shown in Fig 3B, is expressed by (9), where T_BCLGRC_ denotes the propagation delay of the 4-bit BCLGRC shown in Fig 4A. In (9), T_BCLGRC_INT_ specifies the propagation delay of a 4-bit BCLGRC present in an intermediate nibble position of the adder, and T_BCLGRC_LS_ specifies the propagation delay of the least significant 4-bit BCLGRC.

$${FL}_{BCLARC\_RTZ}=T_{XOR3}+(3\times T_{FA})+(6\times T_{BCLGRC\_INT})+T_{BCLGRC\_LS}+T_{Register}$$ (9)

Eq (9) is expanded and given by (10), where T_AO21_, T_AND4_ and T_OR4_ denote the propagation delays of the AO21 complex gate, the 4-input AND gate, and the 4-input OR gate respectively. There is a one-to-one correspondence between the terms present on the right-side of (9) and (10).

$${FL}_{BCLARC\_RTZ}=(T_{CE2}+T_{OR2})+(3\times T_{AO22})+(6\times T_{AO21})+(T_{AO22}+T_{AND4}+T_{OR4}+T_{AO21})+T_{CE2}$$ (10)

The critical datapath traversed for the application of the spacer in the case of the 32-bit QDI BCLARC is highlighted by the red dashed line in Fig 3B. Since the 4-bit QDI BCLGRC shown in Fig 4A is of early output type, and because this is used to construct the QDI BCLARC of Fig 3B, the redundant lookahead carry outputs of all the 4-bit BCLGRCs could assume the spacer simultaneously. But, the redundant lookahead carry output produced by a 4-bit BCLGRC is given as the carry input for the successive 4-bit BCLGRC (or 4-bit BCLG) to produce the corresponding non-redundant lookahead carry output. This carry output then serves as the carry input for the least significant full adder present in the following 4-bit BCLARC (or 4-bit BCLA) to produce the corresponding sum output bit.

With RL_BCLARC_RTZ_ representing the reverse latency of the QDI BCLARC, that corresponds to RTZ handshaking, as shown in Fig 3B, and referring to Fig 5, it is expressed by (11). In (11), T_BCLG_LS_ may be replaced by T_BCLG_INT_ without any loss of generality since the reverse latency would be the same. The expanded version of (11) is given by (12), and there exists a one-to-one correspondence between the terms present on the right-side of (11) and (12).

$${RL}_{BCLARC\_RTZ}=T_{FA}+T_{BCLG\_INT}+T_{BCLG\_LS}+T_{Register}$$ (11)

$${RL}_{BCLARC\_RTZ}=(T_{CE2}+T_{OR2})+(2\times T_{CE2}+T_{OR2})+(T_{AO22}+T_{AND4}+T_{OR4}+T_{AO21})+T_{CE2}$$ (12)

Based on (10) and (12), the forward and reverse latencies of the QDI BCLARC, shown in Fig 3B, which corresponds to RTZ handshaking are calculated to be 1.171ns and 0.849ns, which results in a CT of 2.02ns.

The detailed expressions for forward and reverse latencies corresponding to RTO handshaking are given by (13) and (14). Eqs (13) and (14) are deduced by replacing the propagation delays of the gates mentioned in (10) and (12) with the propagation delays of their dual gates, however, excluding T_CE2_ which is retained as such.

$${FL}_{BCLARC\_RTO}=(T_{CE2}+T_{AND2})+(3\times T_{OA22})+(6\times T_{OA21})+(T_{OA22}+T_{OR4}+T_{AND4}+T_{OA21})+T_{CE2}$$ (13)

$${RL}_{BCLARC\_RTO}=(T_{CE2}+T_{AND2})+(2\times T_{CE2}+T_{AND2})+(T_{OA22}+T_{OR4}+T_{AND4}+T_{OA21})+T_{CE2}$$ (14)

Based on (13) and (14), the forward and reverse latencies of the 32-bit QDI BCLARC (Fig 3B) corresponding to RTO handshaking are calculated to be 1.245ns and 0.933ns, which results in a CT of 2.178ns.

Based on the theoretical calculations of CT, it is noted that the QDI BCLARC architecture achieves 59.1% and 60.2% reductions in CT than the QDI BCLA architecture for a 32-bit addition with respect to RTZ and RTO handshaking respectively. This implies the former (BCLARC) is more beneficial than the latter for performing addition at an enhanced speed. Based on the simulation results obtained, which will be discussed in the next section, it is found that the QDI BCLARC architecture achieves 57% and 55.7% reductions in CT over the QDI BCLA architecture for a 32-bit addition with respect to RTZ and RTO handshaking respectively. Hence, a good correlation is evident between the theoretical calculations and the practical estimates of CT. Although the theoretical calculations of CT may be approximate, nevertheless they are useful as they give a valuable design insight, which is the QDI BCLARC architecture is preferable to the QDI BCLA architecture. Nevertheless, differences between the theoretical calculations and the practical estimates are expected because the interconnect delays and the parasitic are not accounted for in the theoretical calculations of CT.

## 5. Results and discussion

Fifty-six 32-bit QDI and non-QDI (relative-timed) asynchronous adders, which correspond to various architectures such as RCA, CSLA, CCLA, BCLA, BCLARC, and hybrid BCLARC-RCA were physically realized using a 32/28nm CMOS technology [43], including the input registers and the completion detector as shown in Fig 1B. Of the fifty-six asynchronous adders, twenty-eight correspond to RTZ handshaking and a similar number corresponds to RTO handshaking. As mentioned earlier, the 2-input C-element was custom-realized by modifying the AO222 gate to implement the asynchronous adders. A typical-case PVT specification of a high V_t_ standard digital cell library with a recommended supply voltage of 1.05V and an operating junction temperature of 25°C was considered for the implementations and simulations. The registers and completion detectors associated with the asynchronous adders are maintained the same with respect to RTZ and RTO handshaking. This implies the differences between the simulation results of the adders are attributable to the differences between their logic compositions. The default wire load model was considered in the simulations. A virtual clock source was used to constrain the input and output ports of the adders, which did not feature in the adder designs or simulations and hence it does not contribute to the design metrics.

Test benches comprising about two thousand (random) input vectors including data and spacer, which separately correspond to RTZ and RTO handshaking, as used in our prior work [14], were used to verify the functionalities of the adders. The input vectors corresponding to RTZ and RTO handshaking bear a logical equivalence. Functional simulations of all the adders were performed and their respective switching activities were captured which were subsequently used to estimate the average power dissipation.

Synopsys EDA tools were used to estimate the design metrics of the adders. The design metrics estimated include forward and reverse latencies, CT, area, and average power dissipation. The forward latency of an asynchronous circuit is similar to the critical path delay of a synchronous circuit and it is directly estimated. The reverse latency may differ from the forward latency, which is evident from the critical datapaths highlighted in Fig 3A and 3B. The reverse latencies of the asynchronous adders were ascertained from the gate-level timing analysis, and this method was followed for RTZ and RTO handshaking, as done in our previous work [14]. The design metrics of the adders corresponding to RTZ handshaking are given in Table 1, and the design metrics corresponding to RTO handshaking are given in Table 2. Adder legends are provided in the second columns of Tables 1 and 2 to conveniently refer to the individual adders during the discussion. The related literature references pertaining to those adders are also given in Tables 1 and 2. The adders have been grouped according to their architectural type and not according to the chronological order of appearance in the literature.

**Table 1:** Design metrics of several 32-bit asynchronous adders (QDI and non-QDI) corresponding to RTZ handshaking.

Adder Architecture	Adder Legends	Literature Reference	FL^1^ (ns)	RL^2^ (ns)	CT (ns)	Area (μm^2^)	Power (μW)
RCA	Z1	[15]	14.61	14.61	29.22	2529.00	2190
	Z2	[16]^3^	9.26	9.26	18.52	2504.60	2181
	Z3	[17]	9.04	9.04	18.08	2293.14	2172
	Z4	[16]^4^	8.24	8.24	16.48	2423.27	2177
	Z5	[18]	7.00	7.00	14.00	2016.63	2171
	Z6	[19]	4.43	0.58	5.01	2097.96	2174
	Z7	[20]	3.32	0.73	4.05	2049.16	2171
	Z8	[21]	3.10	0.61	3.71	1658.80	2161
	Z9	[22]^5^	2.91	0.62	3.53	1658.80	2168
Uniform CSLA	Z10	[28]	2.46	1.89	4.35	3000.17	2293
Non-uniform CSLA	Z11		3.23	3.23	6.46	3384.44	2312
BCLA	Z12	[25]	3.31	2.93	6.24	2951.88	2191
BCLARC	Z13		2.46	1.69	4.15	2987.46	2192
BCLA	Z14	[25]	3.14	2.88	6.02	2915.29	2188
BCLARC	Z15		2.32	1.68	4.00	2950.87	2189
CCLA	Z16	[26]	2.75	2.75	5.50	2569.65	2177
BCLA	Z17	[27]	3.13	2.88	6.01	2524.92	2178
BCLARC	Z18		2.31	1.67	3.98	2560.50	2179
BCLA	Z19	[14]	2.76	2.50	5.26	2209.78	2174
BCLARC	Z20		2.01	1.38	3.39	2245.36	2176
Hybrid BCLARC-RCA1	Z21		1.93	1.38	3.31	2171.41	2174
Hybrid BCLARC-RCA2	Z22		1.97	1.38	3.35	2097.45	2172
Hybrid BCLARC-RCA3	Z23		2.23	1.38	3.61	2023.49	2170
BCLA	Z24	Proposed	3.46	3.20	6.66	2307.37	2187
BCLARC	Z25		1.76	1.11	2.87	2342.95	2188
Hybrid BCLARC-RCA1	Z26		1.86	1.11	2.97	2256.80	2184
Hybrid BCLARC-RCA2	Z27		2.11	1.11	3.22	2170.64	2181
Hybrid BCLARC-RCA3	Z28		2.36	1.11	3.47	2084.49	2178

^1^ Forward Latency

^2^ Reverse Latency

^3^ Uses strong-indication full adder

^4^ Uses weak-indication full adder

^5^ Uses LOPT_EO_FA of [15] leading to less CT.

**Table 2:** Design metrics of several 32-bit asynchronous adders (QDI and non-QDI) corresponding to RTO handshaking.

Adder Architecture	Adder Legends	Literature Reference	FL^1^ (ns)	RL^2^ (ns)	CT (ns)	Area (μm^2^)	Power (μW)
RCA	O1	[15]	14.15	14.15	28.30	2529.00	2185
	O2	[16]^3^	8.74	8.74	17.48	2374.48	2167
	O3	[17]	8.88	8.88	17.76	2293.15	2168
	O4	[16]^4^	8.03	8.03	16.06	2358.21	2167
	O5	[18]	6.95	6.95	13.90	2016.63	2167
	O6	[19]	3.79	0.56	4.35	2097.96	2170
	O7	[20]	3.31	0.72	4.03	2049.16	2167
	O8	[21]	2.93	0.61	3.54	1658.80	2157
	O9	[22]^5^	2.76	0.61	3.37	1658.80	2164
Uniform CSLA	O10	[28]	2.38	1.85	4.23	3000.17	2285
Non-uniform CSLA	O11		3.15	3.08	6.23	3384.44	2303
BCLA	O12	[25]	3.19	2.86	6.05	2984.41	2184
BCLARC	O13		2.36	1.69	4.05	3019.99	2185
BCLA	O14	[25]	3.10	2.84	5.94	2947.82	2182
BCLARC	O15		2.30	1.67	3.97	2983.40	2183
CCLA	O16	[26]	2.73	2.73	5.46	2553.39	2169
BCLA	O17	[27]	3.06	2.76	5.82	2557.45	2171
BCLARC	O18		2.26	1.66	3.92	2593.03	2172
BCLA	O19	[14]	2.73	2.50	5.23	2193.52	2167
BCLARC	O20		1.95	1.37	3.32	2229.10	2168
Hybrid BCLARC-RCA1	O21		1.88	1.37	3.25	2157.17	2167
Hybrid BCLARC-RCA2	O22		1.89	1.37	3.26	2085.25	2165
Hybrid BCLARC-RCA3	O23		2.13	1.37	3.50	2013.33	2164
BCLA	O24	Proposed	3.38	3.14	6.52	2315.51	2180
BCLARC	O25		1.74	1.15	2.89	2351.09	2181
Hybrid BCLARC-RCA1	O26		1.78	1.15	2.93	2263.92	2178
Hybrid BCLARC-RCA2	O27		2.02	1.15	3.17	2176.74	2175
Hybrid BCLARC-RCA3	O28		2.26	1.15	3.41	2089.57	2172

^1^ Forward Latency

^2^ Reverse Latency

^3^ Uses strong-indication full adder

^4^ Uses weak-indication full adder

^5^ Uses LOPT_EO_FA of [15] leading to less CT.

The area occupancies of various building blocks used to construct the asynchronous adders such as full adders, XOR3 function, 2:1 multiplexers (MUXes), 4-bit CCLA, 4-bit BCLGs and 4-bit BCLGRCs (and 4-bit BCLAs and 4-bit BCLARCs) pertaining to [14–22, 25, 26, 27] and the proposed 4-bit BCLG, BCLGRC, BCLA and BCLARC are given in Table 3, which correspond to RTZ and RTO handshaking. The ‘–’ in Table 3 specifies the non-requirement of the logic block in realizing the corresponding adder.

**Table 3:** Areas of various asynchronous building blocks (in μm^2^) used in diverse adder architectures based on a 32/28nm CMOS process [43].

Adder Legends	Full Adder	XOR3 Logic	2:1 MUX	4-bit CCLA	4-bit BCLG	4-bit BCLGRC	4-bit BCLA	4-bit BCLARC
*RTZ Handshaking*								
Z1	54.64	–	–	–	–	–	–	–
Z2	53.88	–	–	–	–	–	–	–
Z3	47.27	–	–	–	–	–	–	–
Z4	51.34	–	–	–	–	–	–	–
Z5	38.63	–	–	–	–	–	–	–
Z6	41.17	–	–	–	–	–	–	–
Z7	39.65	–	–	–	–	–	–	–
Z8	27.45	–	–	–	–	–	–	–
Z9	27.45	–	–	–	–	–	–	–
Z10, Z11	27.45	–	31.22	–	–	–	–	–
Z12, Z13	41.17	34.56	–	–	113.35	118.43	271.42	276.50
Z14, Z15	39.65	34.56	–	–	113.35	118.43	266.86	271.94
Z16	–	–	–	223.65	–	–	–	–
Z17, Z18	27.45	22.36	–	–	113.35	118.43	218.06	223.14
Z19 to Z23	27.45	22.36	–	–	73.96	79.04	178.67	183.75
Z24 to Z28	27.45	22.36	–	–	86.15	91.24	190.86	195.95
*RTO Handshaking*								
O1	54.64	–	–	–	–	–	–	–
O2	49.81	–	–	–	–	–	–	–
O3	47.27	–	–	–	–	–	–	–
O4	49.30	–	–	–	–	–	–	–
O5	38.63	–	–	–	–	–	–	–
O6	41.17	–	–	–	–	–	–	–
O7	39.65	–	–	–	–	–	–	–
O8	27.45	–	–	–	–	–	–	–
O9	27.45	–	–	–	–	–	–	–
O10, O11	27.45	–	31.22	–	–	–	–	–
O12, O13	41.17	34.56	–	–	117.41	122.50	275.48	280.57
O14, O15	39.65	34.56	–	–	117.41	122.50	270.92	276.01
O16	–	–	–	221.61	–	–	–	–
O17, O18	27.45	22.36	–	–	117.41	122.50	222.12	227.21
O19 to O23	27.45	22.36	–	–	71.92	77.01	176.63	181.72
O24 to O28	27.45	22.36	–	–	87.17	92.25	191.88	196.96

Referring to Table 1, adders Z2 (O2) and Z4 (O4) were constructed using the full adder of [16] which correspond to RTZ (RTO) handshaking. Adders Z1 (O1), Z3 (O3), and Z5 (O5) to Z9 (O9) were constructed using the full adders of [15], [17] and [18] to [22] respectively, which correspond to RTZ (RTO) handshaking. Adders Z1 (O1) to Z9 (O9) correspond to the RCA architecture. Adders Z10 and Z11 (O10 and O11) are CSLAs which were constructed using the early output full adder of [21] and the strong-indication 2:1 MUX of [44], which pertain to RTZ (RTO) handshaking.

Adders Z12 and Z13 (O12 and O13) are BCLAs, constructed using the full adder of [19], the XOR3 function derived from the full adder functionality, and the early output 4-bit BCLG and BCLGRC of [25], which correspond to RTZ (RTO) handshaking. Adders Z14 and Z15 (O14 and O15) are also BCLAs (BCLA and BCLARC respectively), constructed using the full adder of [20], the XOR3 function derived from the full adder functionality, and the early output 4-bit BCLG and BCLGRC of [25], which correspond to RTZ (RTO) handshaking.

Adder Z16 (O16) is a CCLA [26], which pertains to RTZ (RTO) handshaking. Adders Z17 and Z18 (O17 and O18) are also BCLAs (BCLA and BCLARC respectively), constructed using the full adder of [21], the XOR3 function based on the full adder functionality, and the early output 4-bit BCLG and BCLGRC of [25], which correspond to RTZ (RTO) handshaking. Adders Z19 and Z20 (O19 and O20) are also BCLAs (BCLA and BCLARC respectively), constructed using the full adder of [21], the XOR3 function derived from the full adder functionality, and the early output 4-bit BCLG and BCLGRC of [14], which correspond to RTZ (RTO) handshaking. Adders Z21 to Z23 (O21 to O23) are hybrid BCLARC-RCAs, which are derived from Z20 (O20).

Adders Z24 and Z25 (O24 and O25) represent the proposed BCLAs (BCLA and BCLARC respectively), which were realized using the novel 4-bit BCLG and BCLGRC blocks described in Section 4.2, the full adder of [21] and the XOR3 function derived from the full adder functionality, which correspond to RTZ (RTO) handshaking. Adders Z26 to Z28 (O26 to O28) are hybrid BCLARC-RCAs, which are derived from Z25 (O25). It may be seen from Table 3 that some building blocks require the same area for both RTZ and RTO handshaking. For example, the full adder of [15] used to construct Z1 and O1 requires the same area for physical implementation based on RTZ and RTO handshaking. Likewise, the XOR3 function used to construct Z12 to Z15 and O12 to O15 require the same areas for physical realization based on RTZ and RTO handshaking. This is because some of the dual gate equivalents in the digital cell library [43] feature the same area, as remarked in [14] and [20]. For examples, the minimum-size 2-input AND and OR gates of [43] require the same area, which is found to be the case with the gate duals such as AO22 and OA22 gates, and AO222 and OA222 gates. This kind of similar area occupancies by the dual gate equivalents may not be common in all standard digital cell libraries. However, the propagation delay and leakage and dynamic power components of the gates used for RTO handshaking (such as 2-input AND, OA22 and OA222 gates) are generally less than the corresponding metrics of the dual gate equivalents used for RTZ handshaking (such as 2-input OR, AO22 and AO222), as noted from [43]. This explains why the RTO handshaking usually facilitates less delay and power dissipation compared to the RTZ handshaking, as observed from Tables 1 and 2, and validated in [33]. Unfortunately, the physical details about the library components [43] cannot be discussed in detail here due to the proprietary nature of the information.

Referring to the diverse asynchronous adders given in Tables 1 and 2, in terms of area, the RCA architecture is preferable to the CSLA and CLA architectures. This is true even in the case of a synchronous digital design [45, 46]. Hence, from the area perspective, Z8 and O8 are preferable with respect to RTZ and RTO handshaking. Z9 and O9 are discounted as they are non-robust relative-timed RCAs.

As mentioned earlier, CT governs the speed of a QDI or a relative-timed asynchronous circuit that employs delay-insensitive data encoding and a 4-phase handshaking. Among the RCAs, Z9 and O9 report the least CT with respect to RTZ and RTO handshaking. However, Z9 and O9 are relative-timed RCAs which are non-QDI and non-robust, and hence Z8 and O8, which are weak-indication QDI RCAs are preferable among the RCA architecture. Compared to the CTs of Z8 and O8, the CTs of CSLAs (Z10 and Z11 with respect to RTZ handshaking and O10 and O11 with respect to RTO handshaking) and many CLAs (Z12 to Z19 and Z24 with respect to RTZ handshaking and O12 to O19 and O24 with respect to RTO handshaking) are greater. This is mainly due to the substantially reduced reverse latency in the case of Z8 and O8 compared to their respective CSLA and CLA counterparts.

Referring to (10), to process the data, the critical path traversed in the proposed BCLARC (Z25 of Table 1) would involve two 2-input C-elements including a register, seven AO21 gates, four AO22 gates, a 4-input AND gate, a 4-input OR gate and a 2-input OR gate, resulting in a theoretical forward latency of 1.171ns and a practical forward latency of 1.76ns. On the other hand, the critical path traversed in an RCA (say, Z8 of Table 1) to process the data would encounter a register, thirty-two AO22 gates, a 2-input C-element and a 2-input OR gate, resulting in a theoretical forward latency of 2.576ns and a practical forward latency of 3.10ns.

For a similar discussion regarding reverse latency, referring to (12), to process the spacer, the critical path traversed in the proposed BCLARC (Z25) would involve four 2-input C-elements including a register, one AO21 gate, one AO22 gate, a 4-input AND gate, a 4-input OR gate and two 2-input OR gates, resulting in a theoretical reverse latency of 0.849ns and a practical reverse latency of 1.11ns. On the other hand, the critical path traversed in the RCA (Z8 of Table 1) to process the spacer would encounter a register, two AO22 gates, a 2-input C-element and a 2-input OR gate, resulting in a theoretical reverse latency of 0.416ns and a practical reverse latency of 0.61ns. Although the reverse latency of Z8 is less than Z25, the significantly reduced forward latency of Z25 vis-à-vis Z8 compensates to achieve a considerable net reduction in CT for Z25 compared to Z8.

According to the theoretical calculations, the CT of proposed BCLARC (Z25) is 2.02ns and the CT of Z8 is 2.992ns, implying a theoretical reduction in CT by 32.5% for Z25 compared to Z8. According to the practical estimates given in Table 1, Z25 reports a 22.6% reduction in CT than Z8. Similarly, based on the practical estimates, O25, which is the RTO counterpart of Z25, achieves a 18.4% reduction in CT than O8, which is the RTO counterpart of Z8. Overall, the proposed BCLARCs Z25 and O25 feature reduced CTs compared to the CTs of all the other adders in Tables 1 and 2 respectively.

Usually, BCLA architectures incorporating redundant carries tend to have reduced forward and reverse latencies and CT compared to those of plain BCLA architectures which do not have redundant carries, i.e., the QDI BCLARC architecture is preferable to the QDI BCLA architecture in terms of the timing. This observation is already substantiated by the deliberations in Section IV and would be further evident upon comparing Z12 and Z13, Z14 and Z15, Z17 and Z18, Z19 and Z20, and Z24 and Z25 in Table 1, and by comparing O12 and O13, O14 and O15, O17 and O18, O19 and O20, and O24 and O25 in Table 2. Further, this agrees with the observation made in [29] that introducing redundant logic, which can be interpreted as the redundant carry output logic introduced in the BCLARC architecture, which is not available in the BCLA architecture, facilitates overall reductions in the timing.

In the case of CCLAs [26] i.e., Z16 of Table 1 and O16 of Table 2, which are QDI and of early output type, their forward and reverse latencies are equal. This is because the same critical path would be traversed for processing the data and the spacer, and the critical path is data-dependent. Moreover, there is no opportunity for introducing redundant carries in the CCLA architecture to speed-up the carry propagation since the lookahead carry output of say, a 4-bit CCLA is provided as the carry input for the successive 4-bit CCLA in the cascade. As a result, CTs of Z16 and O16 are considerably greater than the CTs of all the BCLARCs. The proposed BCLARC i.e., Z25 achieves a 47.8% reduction in CT compared to Z16. Based on RTO handshaking, O25 achieves a 47.1% reduction in CT compared to O16.

In Tables 1 and 2, hybrid BCLARC-RCAs are also considered. They are denoted by Z21 to Z23 and Z26 to Z28 in Table 1, and O21 to O23 and O26 to O28 in Table 2. A hybrid BCLARC-RCA architecture replaces one or more less significant sub-BCLARC(s) with a similar size RCA, which consists of full adders. For example, Z21 and Z26, Z22 and Z27, and Z23 and Z28 in Table 1 incorporate a 4-bit RCA, an 8-bit RCA and a 12-bit RCA in the least significant adder bit positions as a corresponding replacement for one, two and three instances of a 4-bit BCLARC respectively. While the replacement of one or more 4-bit BCLARCs by a corresponding size RCA could help to reduce the area, it is not guaranteed that such a replacement will always have a beneficial impact on the CT, and rather the contrary might result.

The CTs of Z21, Z22 and Z23, and Z26, Z27 and Z28 given in Table 1 reveal that increasing the size of the sub-RCA in the least significant adder bit positions increases the forward latencies of hybrid BCLARC-RCAs although their reverse latencies remain a constant. The constant reverse latency is because of the traversal of the same critical datapath, shown using the red dashed line in Fig 3B. The forward latencies of Z26, Z27 and Z28, belonging to Table 1, are expressed by (15) to (17). These are obtained by modifying (10) while considering the replacement of sub-BCLARC(s) with a similar sized sub-RCA. To construct the sub-RCA, the QDI early output full adder of [21] was used, and this was used to construct the hybrid BCLARC-RCAs in [14, 27] as well.

$${FL}_{Z26}=(T_{CE2}+T_{OR2})+(3\times T_{AO22})+(6\times T_{AO21})+(5\times T_{AO22})+T_{CE2}$$ (15)

$${FL}_{Z27}=(T_{CE2}+T_{OR2})+(3\times T_{AO22})+(5\times T_{AO21})+(9\times T_{AO22})+T_{CE2}$$ (16)

$${FL}_{Z28}=(T_{CE2}+T_{OR2})+(3\times T_{AO22})+(4\times T_{AO21})+(13\times T_{AO22})+T_{CE2}$$ (17)

By substituting the propagation delays of the gates from [43] in (15), (16) and (17), the theoretical forward latencies of Z26, Z27 and Z28 in Table 1 were calculated to be 1.226ns, 1.451ns and 1.676ns respectively. In Section 4.3.2, the theoretical forward latency of Z25 was calculated to be 1.171ns. Hence, theoretically, Z25 has a reduced forward latency than Z26, Z27 and Z28, which is supported by the practical estimates given in Table 1.

The reverse latency of Z25 was theoretically calculated to be 0.849ns in Section 4.3.2, and the same reverse latency is applicable for Z26, Z27 and Z28 in Table 1. Hence, theoretically, the CTs of Z25, Z26, Z27 and Z28 equate to 2.02ns, 2.075ns, 2.3ns and 2.525ns respectively. This shows that Z25, which is the proposed BCLARC, has a reduced CT than the CTs of Z26, Z27 and Z28, which are the hybrid BCLARC-RCAs. Theoretically, the CT of Z25 is 2.7% less than the CT of Z26, and practically (based on the results given in Table 1), the CT of Z25 is found to be 3.4% less than the CT of Z26. Thus, there is a correlation between the theoretical and practical estimates of CT, and the theoretical calculations tend to provide a valuable design insight.

Based on (15), (16) and (17), and considering the duals of the respective gates with the exception of the 2-input C-elements, the forward latencies of O26, O27 and O28, which are the RTO counterparts of Z26, Z27 and Z28, as mentioned in Table 2, could be theoretically modeled. This can be done by modifying (15), (16) and (17) by replacing the propagation delays of specified gates with the propagation delays of their dual gate equivalents, however, excluding the delay of the 2-input C-element which is retained as such. Theoretically, the forward latencies of O26, O27 and O28 are calculated to be 1.286ns, 1.497ns and 1.708ns respectively. Given that (14) is applicable for O25, O26, O27 and O28, their CTs are theoretically calculated to be 2.178ns, 2.219ns, 2.43ns and 2.641ns. This shows that O25, which represents the RTO equivalent of the proposed BCLARC, has a reduced CT than the CTs of hybrid BCLARC-RCAs viz. O26, O27 and O28. Hence, based on the proposed 4-bit BCLGRCs, portrayed by Figs 4A and 5A, it is inferred that the proposed BCLARC is preferable to the hybrid BCLARC-RCAs on the basis of CT with respect to both RTZ and RTO handshaking.

The proposed BCLARC achieves a substantial reduction in CT compared to the CTs of other BCLARCs and also in comparison with the optimum CT of a hybrid BCLARC-RCA, reported in the latest work [14]. Hence, hybrid BCLARC-RCAs corresponding to [25, 27] were not considered as they would be sub-optimum.

With respect to power dissipation, almost all the asynchronous adders, whether they are QDI or non-QDI, dissipate quite nearly the same power with the standard deviation from the mean of the power dissipation estimated to be 33.5 for RTZ handshaking (Table 1) and 33.1 for RTO handshaking (Table 2). The small values of standard deviations are because all the asynchronous adders mentioned in Tables 1 and 2 embed the monotonic cover constraint, discussed in Section 3.2. Hence, the power dissipation of QDI and non-QDI (relative-timed) adders do not vary considerably and are confined to small ranges of 2161μW– 2312μW in the case of Table 1, and 2157μW– 2303μW in the case of Table 2.

**Fig 6:**
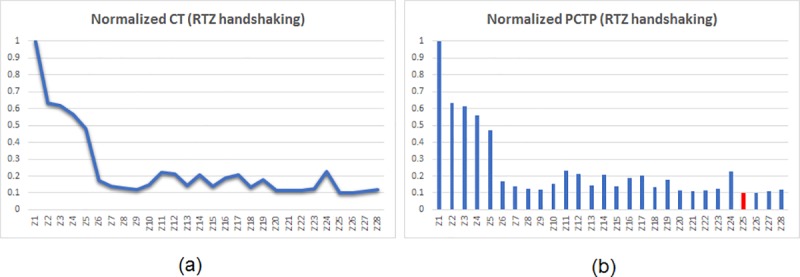
Plots of normalized values of (a) CT and (b) PCTP of several 32-bit asynchronous adders corresponding to RTZ handshaking. The adder legends are referenced in Table 1. The red bar in (b) corresponds to the proposed 32-bit BCLARC (Z25) which is energy-efficient than the rest.

**Fig 7:**
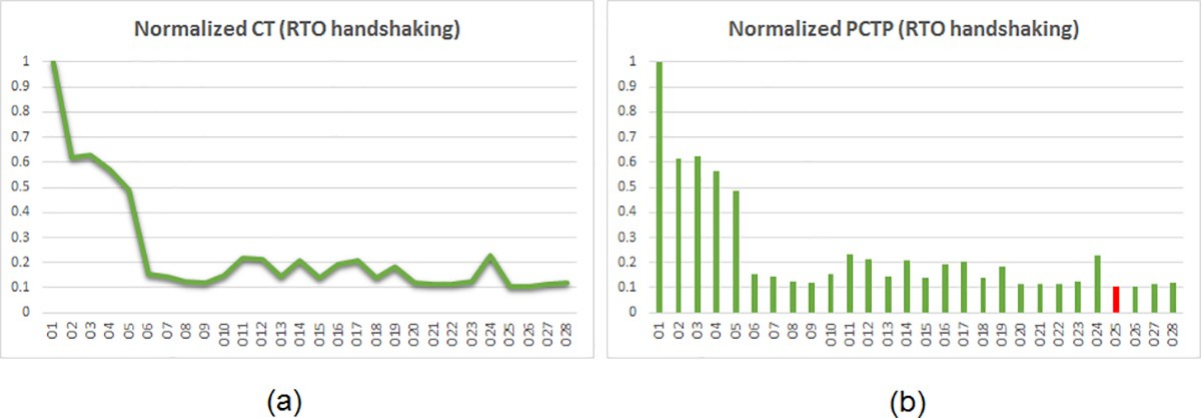
Plots of normalized values of (a) CT and (b) PCTP of several 32-bit asynchronous adders corresponding to RTO handshaking. The adder legends are referenced in Table 2. The red bar in (b) corresponds to the proposed 32-bit BCLARC (O25) which is energy-efficient than the rest.

PCTP governs the low power/energy aspect. The PCTPs of the asynchronous adders were calculated and normalized. The normalization was performed such that the highest PCTP among the set of asynchronous adders corresponding to a particular handshake protocol was normalized to 1, and the actual PCTPs of the remaining adders were divided by the highest PCTP. Thus, after normalization, the least value of PCTP reflects the optimum low power/energy design. The plots of normalized CT and PCTP values corresponding to RTZ handshaking are shown side-by-side in Fig 6A and 6B, and the similar plots for RTO handshaking are portrayed by Fig 7A and 7B.

Given that the average power dissipation of all the asynchronous adders is quite nearly the same, it may be observed that the differences in their PCTP are mainly due to the differences in their CTs. In other words, CT mainly influences the PCTP of the asynchronous adders. This may be evident upon perusing Fig 6A and 6B, and also Fig 7A and 7B.

## 6. Conclusions

This article presented a new QDI early output sub-BCLG/BCLGRC that forms the basis for constructing a QDI early output BCLA/BCLARC. In particular, we discussed the design of a 4-bit QDI BCLA and a 4-bit QDI BCLARC which serve as the building blocks for constructing the QDI early output BCLARC. For an example, we considered a 32-bit addition and compared the proposed QDI BCLARC with several asynchronous adders, which are QDI and non-QDI (relative-timed). Further, hybrid BCLARC-RCAs were considered for the comparison. The simulation results show that the proposed QDI early output BCLARC (Z25 of Table 1 and O25 of Table 2) is efficient in terms of speed (CT) as well as low power/energy (PCTP).

With respect to RTZ handshaking, the proposed QDI BCLARC (Z25 of Table 1) achieves the following reductions in design metrics over its counterparts for 32-bit addition: i) 22.6% and 21.7% reductions in CT and PCTP respectively compared to an optimum QDI early output RCA (i.e., Z8), ii) 18.7% and 17.9% reductions in CT and PCTP respectively compared to an optimum relative-timed RCA (i.e., Z9), iii) 34% and 37% reductions in CT and PCTP respectively compared to an optimum uniform input-partitioned QDI early output CSLA (i.e., Z10), iv) 47.8% and 47.6% reductions in CT and PCTP respectively compared to an optimum QDI early output CCLA (i.e., Z16), v) 45.4% and 45.1% reductions in CT and PCTP respectively compared to an optimum QDI early output BCLA (i.e., Z19), vi) 15.3% and 14.9% reductions in CT and PCTP respectively compared to an optimum QDI early output BCLARC (i.e., Z20), and vii) 13.3% and 12.7% reductions in CT and PCTP respectively compared to an optimum QDI early output hybrid BCLARC-RCA (i.e., Z21).

Based on RTO handshaking, the proposed QDI BCLARC (O25 of Table 2) achieves the following reductions in design metrics over its counterparts for 32-bit addition: i) 18.4% and 17.5% reductions in CT and PCTP respectively compared to an optimum QDI early output RCA (i.e., O8), ii) 14.2% and 13.6% reductions in CT and PCTP respectively compared to an optimum relative-timed RCA (i.e., O9), iii) 31.7% and 34.8% reductions in CT and PCTP respectively compared to an optimum uniform input-partitioned QDI early output CSLA (i.e., O10), iv) 47.1% and 46.8% reductions in CT and PCTP respectively compared to an optimum QDI early output CCLA (i.e., O16), v) 44.7% and 44.4% reductions in CT and PCTP respectively compared to an optimum QDI early output BCLA (i.e., O19), vi) 13% and 12.4% reductions in CT and PCTP respectively compared to an optimum QDI early output BCLARC (i.e., O20), and vii) 11.1% and 10.5% reductions in CT and PCTP respectively compared to an optimum QDI early output hybrid BCLARC-RCA (i.e., O21).

Further work would be to investigate the usefulness of the proposed QDI BCLARC in realizing other computer arithmetic operations of practical significance within the realms of accurate and approximate computing.

## References

[1] Available from: https://irds.ieee.org/roadmap-2017 (last accessed on 19 November 2018).

[2] Tang BZ, Lane F, Low power QDI asynchronous FFT. In: Proceedings of the 22nd IEEE International Symposium on Asynchronous Circuits and Systems (ASYNC); 2016. pp. 87–88.

[3] van Berkel CH, Josephs MB, Nowick SM. Applications of asynchronous circuits. Proceedings of the IEEE. 1999;87: 223–233. 10.1109/5.740016

[4] Martin AJ, Nystrom M. Asynchronous techniques for system-on-chip design. Proceedings of the IEEE. 2006;94: 1089–1120. 10.1109/JPROC.2006.875789

[5] Kundu S, Sreedhar A. Nanoscale CMOS VLSI circuits: Design for manufacturability. New York, USA: McGraw-Hill; 2010.

[6] Bouesse G, Sicard G, Baixas A, Renaudin M, Quasi delay insensitive asynchronous circuits for low EMI. In: Proceedings of the 4th International Workshop on Electromagnetic Compatibility of Integrated Circuits (EMC Compo); 2004. pp. 27–31.

[7] Plana LA, Riocreux PA, Bainbridge WJ, Bardsley A, Temple S, Garside JD et al SPA–a secure amulet core for smartcard applications. Microprocessors and Microsystems. 2003;27: 431–446. 10.1016/S0141-9331(03)00093-0

[8] Renaudin M, Monnet Y, Asynchronous design: fault robustness and security characteristics. In: Proceedings of the 12th IEEE International On-Line Testing Symposium (IOLTS); 2006. pp. 1–4.

[9] Sparsø J, Furber S. Principles of asynchronous circuit design: A systems perspective. Dordrecht: Kluwer Academic Publishers; 2001.

[10] Martin AJ, Can asynchronous techniques help the SoC designer?. In: Proceedings of the IFIP International Conference on Very Large Scale Integration (VLSI-SoC); 2006. pp. 7–11.

[11] Chang K-L, Chang JS, Gwee B-H, Chong K-S. Synchronous-logic and asynchronous-logic 8051 microcontroller cores for realizing the internet of things: a comparative study on dynamic voltage scaling and variation effects. IEEE Journal on Emerging and Selected Topics in Circuits and Systems. 2013;3: 23–34. 10.1109/JETCAS.2013.2243031

[12] Martin AJ, The limitation to delay-insensitivity in asynchronous circuits. In: Proceedings of the 6th MIT Conference on Advanced Research in VLSI; 1990. pp. 263–278.

[13] Martin AJ, Prakash P, Asynchronous nano-electronics: preliminary investigation. In: Proceedings of the 14th IEEE International Symposium on Asynchronous Circuits and Systems (ASYNC); 2008. pp. 58–68.

[14] Balasubramanian P, Maskell D, Mastorakis N. Low power robust early output asynchronous block carry lookahead adder with redundant carry logic. Electronics. 2018;7: 1–21, Article #243. 10.3390/electronics7100243

[15] Singh NP. A design methodology for self-timed systems. M.Sc. Thesis, Massachusetts Institute of Technology. 1981. Available from: https://pdfs.semanticscholar.org/c019/0d6b135d409142a8703c6188284020a0c59b.pdf?_ga=2.184603823.789291288.1553256324-1229169596.1553256324

[16] Sparsø J, Staunstrup J. Delay-insensitive multi-ring structures. Integration, the VLSI Journal. 1993;15: 313–340. 10.1016/0167-9260(93)90035-B

[17] Toms WB. Synthesis of quasi-delay-insensitive datapath circuits. Ph.D. Thesis, The University of Manchester. 2006. Available from: http://apt.cs.manchester.ac.uk/ftp/pub/amulet/theses/Toms06_phd.pdf

[18] Folco B, Bregier V, Fesquet L, Renaudin M, Technology mapping for area optimized quasi delay insensitive circuits. In: Proceedings of the IFIP 13th International Conference on Very Large Scale Integration (VLSI-SoC); 2005. pp. 146–151.

[19] Balasubramanian P, Edwards DA, A delay efficient robust self-timed full adder. In: Proceedings of the IEEE 3rd International Design and Test Workshop (IDT); 2008. pp. 129–134.

[20] Balasubramanian P. A latency optimized biased implementation style weak-indication self-timed full adder. Facta Universitatis, Series: Electronics and Energetics. 2015;28: 657–671. 10.2298/FUEE1504657B

[21] Balasubramanian P. A robust asynchronous early output full adder. WSEAS Transactions on Circuits and Systems. 2011;10: 221–230.

[22] Balasubramanian P, Yamashita S. Area/latency optimized early output asynchronous full adders and relative-timed ripple carry adders. SpringerPlus. 2016;5:440: 1–26. 10.1186/s40064-016-2074-z 27104128 PMC4828369

[23] Stevens KS, Ginosar R, Rotem S. Relative timing, IEEE Transactions on VLSI Systems. 2003;11: 129–140. 10.1109/TVLSI.2002.801606

[24] Cheng F-C, Unger SH, Theobald M. Self-timed carry-lookahead adders. IEEE Transactions on Computers. 2000;49: 659–672. 10.1109/12.863035

[25] Balasubramanian P, Edwards DA, Toms WB. Self-timed section-carry based carry lookahead adders and the concept of alias logic. Journal of Circuits, Systems, and Computers. 2013;22: 1350028–1–1350028–24. 10.1142/S021812661350028X

[26] Balasubramanian P, Dhivyaa D, Jayakirthika JP, Kaviyarasi P, Prasad K, Low power self-timed carry lookahead adders. In: Proceedings of the 56th IEEE International Midwest Symposium on Circuits and Systems (MWSCAS); 2013. pp. 457–460.

[27] Balasubramanian P, Dang C, Maskell DL, Prasad K, Asynchronous early output section-carry based carry lookahead adder with alias carry logic. In: Proceedings of the IEEE 30th International Conference on Microelectronics (MIEL); 2017. pp. 293–298.

[28] Balasubramanian P. Asynchronous carry select adders. Engineering Science and Technology, an International Journal. 2017;20: 1066–1074. 10.1016/j.jestch.2017.02.003

[29] Balasubramanian P, Edwards DA, Toms WB. Redundant logic insertion and latency reduction in self-timed adders. VLSI Design. 2012;2012: 1–13, Article ID 575389. 10.1155/2012/575389

[30] Muller DE, Bartky WS, A theory of asynchronous circuits. In: Proceedings of an International Symposium on the Theory of Switching; 1959. Part I, pp. 204–243.

[31] Bose B. On unordered codes. IEEE Transactions on Computers. 1991;40: 125–131. 10.1109/12.73583

[32] Piestrak SJ, Nanya T, Towards totally self-checking delay-insensitive systems. In: Proceedings of the 25th International Symposium on Fault-Tolerant Computing (FTCS); 1995. pp. 228–237.

[33] Moreira MT, Guazzelli RA, Calazans NLV, Return-to-one protocol for reducing static power in C-elements of QDI circuits employing m-of-n codes. In: Proceedings of the 25th Symposium on Integrated Circuits and Systems Design (SBCCI); 2012. pp. 1–6.

[34] Seitz CL. System timing In: Mead C, Conway L, editors. Introduction to VLSI systems. Reading, Massachusetts; 1980 pp. 218–262.

[35] Brej C. Early output logic and anti-tokens. Ph.D. Thesis, The University of Manchester. 2006. Available from: http://apt.cs.manchester.ac.uk/ftp/pub/amulet/theses/Brej06_phd.pdf

[36] Balasubramanian P, Mastorakis NE, QDI decomposed DIMS method featuring homogeneous/heterogeneous data encoding. In: Proceedings of the International Conference on Computers, Digital Communications and Computing (ICDCC); 2011. pp. 93–101.

[37] Balasubramanian P. Comments on “Dual-rail asynchronous logic multi-level implementation”. Integration, the VLSI Journal. 2016;52: 34–40. 10.1016/j.vlsi.2015.08.001

[38] Varshavsky VI. Self-timed control of concurrent processes: The design of aperiodic logical circuits in computers and discrete systems. (Translated from the Russian by Yakovlev AV). Dordrecht: Kluwer Academic Publishers; 1990 pp. 77–85.

[39] Balasubramanian P, Arisaka R, Arabnia HR, RB_DSOP: a rule based disjoint sum of products synthesis method. In: Proceedings of the 12th International Conference on Computer Design (CDES); 2012. pp. 39–43.

[40] Weinberger A, Smith JL. A logic for high-speed addition. National Bureau of Standards Publications. 1958;591: 3–12.

[41] Omondi AR. Computer arithmetic systems: Algorithms, architecture and implementations. London: Prentice Hall International (UK) Limited; 1994.

[42] Balasubramanian P. Comparative evaluation of quasi-delay-insensitive asynchronous adders corresponding to return-to-zero and return-to-one handshaking. Facta Universitatis, Series: Electronics and Energetics. (Invited Paper). 2018;31: 25–39. 10.2298/FUEE1801025B

[43] Synopsys SAED_EDK32/28_CORE Databook, Revision 1.0.0, 2012.

[44] Balasubramanian P, Edwards DA, Power, delay and area efficient self-timed multiplexer and demultiplexer designs. In: Proceedings of the IEEE 4th International Conference on Design and Technology of Integrated Systems in Nanoscale Era (DTIS); 2009. pp. 173–178.

[45] Nagendra C, Owens RM, Irwin MJ. Power-delay characteristics of CMOS adders. IEEE Transactions on VLSI Systems. 1994;2: 377–381. 10.1109/92.311649

[46] Balasubramanian P. Performance comparison of some synchronous adders. arXiv preprint, arXiv:1810.01115; 2018. pp. 1–9. Available from: https://arxiv.org/ftp/arxiv/papers/1810/1810.01115.pdf (last accessed on 18 May 2019).

